# Use of a language intervention to reduce vaccine hesitancy

**DOI:** 10.1038/s41598-021-04249-w

**Published:** 2022-01-07

**Authors:** Janet Geipel, Leigh H. Grant, Boaz Keysar

**Affiliations:** The Department of Psychology, The University of Chicago, 5848 South University Avenue, Chicago, IL 60637 USA

**Keywords:** Psychology, Human behaviour

## Abstract

Vaccine hesitancy is a major global challenge facing COVID-19 immunization programs. Its main source is low public trust in the safety and effectiveness of the vaccine. In a preregistered experimental study, we investigated how using a foreign language when communicating COVID-19 vaccine information influences vaccine acceptance. Hong Kong Chinese residents (*N* = 611) received COVID-19 vaccine information either in their native Chinese or in English. English increased trust in the safety and effectiveness of the vaccine and, as a result, reduced vaccine hesitancy. This indicates that language can impact vaccine attitudes and demonstrate the potential of language interventions for a low cost, actionable strategy to curtail vaccine hesitancy amongst bilingual populations. Language interventions could contribute towards achieving the United Nations Sustainable Development Goal of health and well-being.

## Introduction

The World Health Organization lists vaccine hesitancy as one of the top ten threats to global health^1^. Defined as the delay or outright refusal of vaccination despite its availability, vaccine hesitancy has led to new outbreaks of diseases such as polio and measles that have been under control for decades. And it has increasingly become a global issue, crossing national and socioeconomic boundaries and impacting the well-being of millions of people worldwide^2,3^. With the COVID-19 pandemic, vaccine hesitancy has become an acute problem, following a year in which the entire world has struggled with the impact of the pandemic, which has caused more than 3 million deaths worldwide^4^. While newly developed COVID-19 vaccines are highly effective and could help save millions of lives^5^, a certain vaccination rate must be reached in order to curtail widespread transmission. It is therefore crucial to develop evidence-based ways to reduce vaccine hesitancy. Here we examine a low cost and actionable strategy towards this goal: providing vaccine information in a foreign, as opposed to a native, language.

We conducted the study in Hong Kong from March 27th to April 12th, 2021, when vaccination uptake was relatively low and the reported vaccine hesitancy was high. While there are debates regarding the minimum vaccination rate for herd immunity, the estimated threshold varies from 55 to 85% of the population^6–8^. Yet surveys suggest widespread vaccine hesitancy, with some of the lowest rates of vaccine acceptance reported in Hong Kong, where only 37.2% of citizens express willingness to get a COVID-19 vaccine^9,10^. Even amongst health workers in Hong Kong, vaccine acceptance was low ranging between 40.0 and 63.0%^11,12^. If this high vaccine hesitancy persists, it would reduce the likelihood of achieving herd immunity and preventing COVID-19 outbreaks.

One of the main reasons for vaccine hesitancy is lack of public trust in the purported safety and effectiveness of the vaccine^10,13,14^. Therefore, in order to develop effective COVID-19 vaccine campaigns, it is important to find ways to increase public trust in the vaccine. We examined one such method with Hong Kong residents, namely, communicating vaccine information in English rather than in their native Chinese.

Irrespective of what drives the mistrust and negativity towards the vaccine amongst Hong Kong Chinese residents, these attitudes have been developed and reinforced predominantly in their native language context, Chinese. When people encode information and experiences in memory, they keep a trace of the linguistic context^15^. The presence of the same linguistic context during retrieval facilitates the retrieval of the associated memory. Because people more readily recall information when shared through the language it was initially encoded^15^, we expected that the mistrust and negativity towards COVID-19 vaccines will loom larger when the vaccine information is provided in the participants’ native Chinese than in their foreign English. Thus, we anticipated that English would attenuate mistrust and negativity towards the vaccine and consequently increase the intention to vaccinate.

Furthermore, foreign language use can decrease negativity towards novel technologies and products. Native Italian speakers judged technologies, such as nanotechnology and biotechnology, overall more positively when these were presented in their foreign English than in their native Italian^16^. Furthermore, native Italian, German, and Dutch speakers were more willing to consume sustainable but aversive products, such as mealworm food and recycled wastewater, when these were described in a foreign language rather than in their native tongue^17^. The foreign language promoted more positive feelings towards these products, which resulted in higher acceptance.

Other studies have also shown that how people feel about novel products is driven by differences in trust^18,19^. For example, enhanced social trust increased positive feelings and decreased negative feelings towards the novel avian flu vaccine, which, in turn, increased intentions to get the vaccine^18^. Given that trust and feelings are closely related concepts^19^, and that people particularly rely on trust when judging things that are novel to them^20^, these results raise the possibility that communicating COVID-19 vaccine information in a foreign language might reduce mistrust in the vaccine and, therefore, decrease vaccine hesitancy.

Hong Kong Chinese bilinguals provided an ideal opportunity to test this theory for a couple of reasons. First, COVID-19 vaccine hesitancy was relatively high in Hong Kong compared to other countries^10,12,21^. Second, Chinese and English are official languages in Hong Kong and, hence, many government and healthcare resources are readily available in both languages. Therefore, the language manipulation represents an actionable intervention to reduce COVID-19 vaccine hesitancy in Hong Kong.

To study the impact of foreign language use, we provided COVID-19 vaccine information to unvaccinated Hong Kong residents and randomly assigned them to receive the information either in their native Chinese or in English. We then asked them whether they intend to get the vaccine and how much they trust the vaccine, among other questions.

## Results

The data, analysis script and materials are publicly available online on *Open Science Framework*. The study design and number of participants was preregistered on www.AsPredicted.org.

### Descriptive data of vaccine hesitancy

**Figure 1:**
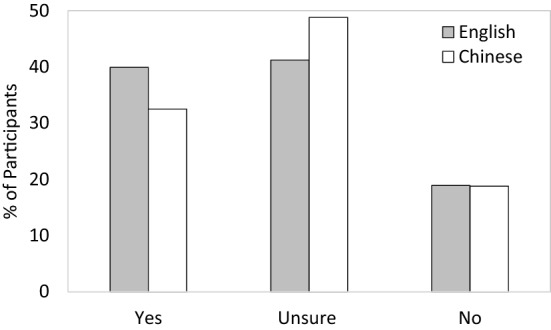
Intention to vaccinate by language condition.

Dovetailing with existing research, we found a high degree of COVID-19 vaccine hesitancy. Out of the 611 participants, only 36.0% (220) said they plan to get vaccinated, 45.2% (276) indicated that they were unsure, and 18.8% (115) indicated that they would not get vaccinated. Most importantly, vaccine hesitancy depended on language as we predicted. The use of English reduced vaccine hesitancy, with more people saying they intend to get vaccinated in the English (39.9%) than in the Chinese (32.5%) condition, and fewer saying they are unsure in English (41.2%) than in Chinese (48.8%). Language did not impact the rate of outright refusal (“No”: English: 18.9%, Chinese: 18.8%). In sum, the foreign language English helped turn hesitancy into acceptance (see Fig. 1).

### Predicting vaccine hesitancy by language

Our main interest was to examine whether language affects vaccine hesitancy. Hence, we grouped responses into ones that indicated no hesitancy (0 = *Yes*) and ones that indicated hesitancy or refusal (1 = *Unsure*, 1 = *No*). A binary logistic regression was conducted examining the dichotomous COVID-19 vaccine hesitancy variable as a function of language (0 = *Chinese*, 1 = *English*), gender (0 = *female*, 1 = *male*), age, education, and general health. The logistic regression model was statistically significant, χ^2^(5) = 27.98, *P* < 0.001. The model correctly classified 62.4% of the cases. As anticipated, language accounted for a significant proportion of variance in vaccine hesitancy (*B* = 0.41, Wald = 5.43, *P* = 0.020, odds ratio = 0.67). Participants reading the COVID-19 vaccine information in English were less hesitant about getting the vaccine (*Mean* = 0.60) than were those reading the same information in their native Chinese (*Mean* = 0.68). Gender also accounted for significant variance in vaccine hesitancy (*B* = 0.54, Wald = 9.19, *P* = 0.002, odds ratio = 1.71), with female participants being less hesitant (*Mean* = 0.30) than male participants (*Mean* = 0.42). Age was also related to vaccine hesitancy (*B* = − 0.02, Wald = 7.01, *P* = 0.008, odds ratio = 0.98), with younger participants being less hesitant (*Median* = 0.34, *Mean* = 0.38, *SD* = 13.90) than older participants (*Median* = 0.39, *Mean* = 0.40, *SD* = 12.61). Finally, general health also influenced vaccine hesitancy (*B* = − 0.22, Wald = 5.77, *P* = 0.016, odds ratio = 0.80), with participants indicating poorer health being less hesitant (*Mean* = 0.29, *SD* = 0.64) than participants indicating better health (*Mean* = 0.30, *SD* = 0.42). Education did not significantly influence rates of vaccine hesitancy (*B* = − 0.23, Wald = 2.52, *P* = 0.112, odds ratio = 0.80).

### Explaining the language effect on vaccine hesitancy with trust in the vaccine

**Figure 2:**
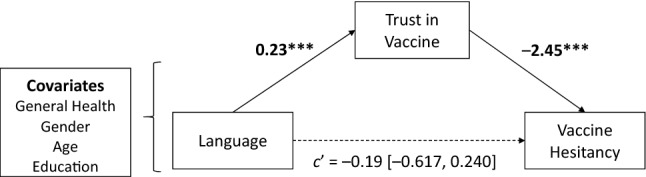
Language effect on vaccine hesitancy explained by trust in the COVID-19 vaccine. Mediation coefficients represent unstandardized coefficients (95% CI in brackets).

We expected that communicating COVID-19 vaccine information in English would increase trust in the vaccine and, through this, reduce vaccine hesitancy. As predicted, trust in the vaccine was significantly higher in English (*M* = 3.22, *SD* = 0.78) than in Chinese (*M* = 3.04, *SD* = 0.91), Welch’s *F*(1, 606.55) = 7.55, *P* = 0.006, *d* = 0.22. We used the mean score across the two trust measures as they were highly correlated (*r*[609] = 0.787, *P* < 0.001). To test whether increased trust reduced hesitancy in English we employed a mediation model using 10,000 bootstrapping samples and computing the 95% confidence intervals (see Fig. 2).

As predicted, the effect of language on vaccine hesitancy was mediated by trust in the vaccine, controlling for general health, gender, age and education (indirect effect: *b* = − 0.56, 95% CI [− 0.915, − 0.248]). The effect of language on vaccine hesitancy was reduced when controlling for trust (direct effect: *b* = − 0.19, 95% CI [− 0.617, 0.240]), consistent with full mediation. Trust remains a significant mediator when the covariates (general health, gender, age, and education) are omitted from the model (indirect effect: − 0.45, 95% CI [− 0.805, − 0.129]; direct effect: *b* = − 0.18, 95% CI [− 0.605, 0.231]).

## Discussion

Vaccine hesitancy presents a major barrier to improving health and well-being around the globe. We investigated how the language used to communicate COVID-19 vaccine information influences vaccine hesitancy. We provide evidence that the use of a foreign language increased trust in the safety and effectiveness of the vaccine compared to identical information communicated in the native language. In turn, the higher trust associated with the foreign language reduced COVID-19 vaccine hesitancy. These findings suggest that feelings of trust when making health decisions depend not only on the content of health information but also on the nature of the language used to communicate it.

Studies have shown that foreign language use can influence judgment and decision making in different domains including risk taking^22^ and morality^23^. Here we demonstrate that language can also influence an extremely consequential health decision, namely whether to get vaccinated during a pandemic. Research has further suggested that language is a powerful social cue that can influence trust^24^. We showed that the language used in communications can influence trust and, as a result, the decision to vaccinate.

Because English was a foreign language for our participants, they might have experienced more disfluency when comprehending the information in English than in Chinese. This could have influenced perceived trust in the vaccine and consequently willingness to be vaccinated. However, this account would predict the opposite of what we found. Disfluency decreases trust rather than increases it^25^. For example, in the “trust game” players show lower trust in people with disfluent names than in those with fluent names^26^. Hence, if English communication is more disfluent, then it should have prompted lower trust in the vaccine and thus increased vaccine hesitancy. However, the opposite was true.

The impact of language on vaccine hesitancy should depend on how it affects trust. Here we showed that when the native language context is associated with relatively low trust in the vaccine, the use of a foreign language increases trust and, therefore, reduces vaccine hesitancy. But in situations where the native language is associated with higher trust than the foreign language, we would expect the opposite. For example, consider the case of first generation immigrant communities such as Arab immigrants in Europe. For such communities, trust in the information that is provided in their native tongue, Arabic, might be higher than in information provided in the local language. In such cases, communications through the foreign language would be predicted to lead to lower trust in the vaccine, thereby increasing vaccine hesitancy. In this sense, language interventions should consider local conditions by understanding how each language impacts trust.

Other determinants of trust associated with a given language could be further explored in the service of reducing vaccine hesitancy. For example, it is possible that when one language of bilinguals has a higher status, people will trust the information provided in it more. In Hong Kong, English does not have higher status than Cantonese^27^, which suggests that the effect we found is not a function of differential language status. Yet in situations where one language has a higher status than the other, the higher status language may increase trust in the information thereby reducing hesitancy.

### Limitations

This study examined a particular population, and a specific native-foreign language combination. It would be important to further investigate the generalizability of the current findings in different populations and with different native-foreign language combinations. Furthermore, the intervention that we identified only applies to bilingual populations. However, estimates show that more than half of the global population uses two or more languages in everyday life^28^, suggesting that this language intervention could be widely actionable. In monolingual populations, other language interventions could be explored, such as using a dialect towards which people have positive attitudes that might lead to higher trust^29^ and reduced vaccine hesitancy.

## Conclusion

We provide evidence for a low cost and actionable language intervention to reduce vaccine hesitancy amongst Hong Kong Chinese residents. Such language interventions can influence other health decisions and extend to other cultures. However, the selection of language should consider the local conditions. In cases where the native language context is associated with low trust, the use of a foreign language can enhance trust and reduce vaccine hesitancy. In cases where the foreign language is associated with low trust, the native language should be preferred. Public health campaigns therefore could use such language interventions strategically to boost vaccination uptake and other beneficial preventative behaviors such as cancer screening. Such language strategies can promote the United Nations Sustainable Development Goal 3 of “good health and well-being”^30^.

## Methods

The study design, sample size and materials were preregistered on www.AsPredicted.org. The data and study materials are available online in the Supplementary Materials and on https://osf.io/bdhvx/?view_only=02c423439eac40af8a9a57c580bb0588. All participants provided written informed consent prior to participation. All procedures were approved by the Social and Behavioral Sciences Institutional Review Board at the University of Chicago. All methods were carried out in accordance with the Declaration of Helsinki.

### Participants

We recruited 624 native Cantonese adult speakers (50.0% male, 49.4% female, 0.6% non-binary, *M*_age_ = 38.7 years, *SD*_age_ = 13.4, age range 18 to 92 years) through the University of Chicago Francis and Rose Yuen Campus in Hong Kong. Participants were invited to complete a survey for a compensation of a HK$50 voucher. Participants were eligible to take part in the study if they were (a) native Cantonese speakers, (b) did not grow up speaking English at home, (c) started to learn English formally at age 3 or later, (d) did not receive a COVID-19 vaccine yet, and (e) had English proficiency of at least good and a Cantonese proficiency of at least very good or native-speaker ability (1 = *poor*, 2 = *fair*, 3 = *good*, 4 = *very good*, 5 = *native-speaker ability*). As preregistered, we excluded 11 participants (1.8%) because they failed comprehension checks by incorrectly translating two or three key sentences of the materials (out of three). Furthermore, we excluded two participants (0.3%) because they indicated having low Cantonese language proficiency (< 3 mean score across four scales, 1 = *poor* to 5 = *native level*).

The reported results are based on the remaining 611 participants (49.9% male, 49.4% female, 0.7% non-binary, *M*_age_ = 38.7, *SD*_age_ = 13.5, age range: 18 to 92 years). Of these, 320 participants were randomly assigned to the native Chinese condition (53.8% male, 45.6% female, *M*_age_ = 39.4, *SD*_age_ = 13.5, age range: 18 to 75 years), and 291 participants to the foreign English condition (45.7% male, 53.6 female, *M*_age_ = 38.1, *SD*_age_ = 13.4, age range: 19 to 92 years). On average, participants reported to have an intermediate proficiency level of English (*M* = 3.41, *SD* = 0.76, 95% CI [3.35, 3.47]) and started to learn English in a formal context by the age of four (95% CI [4.76, 5.19]).

### Materials and measures

Participants read about why they should get the vaccine, how the vaccine works, and possible side effects of the vaccine in either their native Chinese or English adapted from the Hong Kong Department of Health (for the full descriptions see Table S1 in the Supplementary Materials available online). At the time of the study, Hong Kong residents could select the type of vaccine with which to be inoculated, so we did not mention a specific vaccine in the information^31^. We measured intention to vaccinate by asking participants, “If a vaccine that protects you from COVID-19 disease was available free of charge, would you get it?” (*Yes*, *Unsure*, *No*). We also asked participants to evaluate their trust in the effectiveness and safety of the vaccine, “Overall, how much do you trust that the COVID-19 vaccine will be effective?” and “Overall, how much do you trust that the COVID-19 vaccine will be safe?” (1 = *Do not trust at all*, 2 = *Hardly trust*, 3 = *Trust a little*, 4 = *Mostly trust*, 5 = *Completely trust*). Furthermore, we collected a number of exploratory measures of secondary interest (see Supplementary Information for the full set). In order to ensure that all participants had a sufficient proficiency in English to understand the materials, at the end of the study all participants, regardless of language condition, were asked to translate three key sentences from English to Chinese. Finally, participants were asked about their general health, age, gender, and education level.

## Supplementary Information

Supplementary Information.
