# Supplementary material for: Use of a language intervention to reduce vaccine hesitancy
Source: Sci Rep. 2022 Jan 7;12:253. doi: 10.1038/s41598-021-04249-w (PMC8742025; doi:10.1038/s41598-021-04249-w)
Supplement: Supplementary file 1 — Supplementary Information. [file 41598_2021_4249_MOESM1_ESM.pdf]

Supplementary Materials for

Use of a Language Intervention to Reduce Vaccine Hesitancy

Janet Geipel, Leigh H. Grant, & Boaz Keysar

Correspondence to: jgeipel@uchicago.edu

This PDF file includes:

Methods and Materials
Tables S1 to S4
Secondary Results
Figures S1 to S2

Methods

Power Analysis

We determined the number of participants by conducting two a-priori power analyses, one for a chi-square test and one for a simple one-way analysis of variance (*F*-test). For the chi-square test, we used the following estimates: effect size w = 0.15 (small to medium, estimated), α = .05, power = .95, *df* = 1. This analysis suggested a minimum of 578 participants. For the *F*-test, we used the following estimates: effect size f = 0.15 (small to medium, estimated), α = .05, power =.95, number of groups = 2 (language: foreign, native). This analysis suggested a minimum of 580 participants. We preregistered a total sample of 600 participants to prevent reduction in statistical power due to anticipated exclusions.

Materials

Participants were randomly assigned to either the native Chinese or foreign English condition and saw all information in the assigned language. Participants were welcomed using the following information, "*Welcome! In this study we ask your opinion about the vaccine that can protect you from the coronavirus disease 2019 (COVID-19)*". Then participants were asked to respond to a series of pre-screen questions based on our inclusion criteria (see full text). If they passed the pre-screen, they saw the consent form followed by the instruction, "*You will see information about the COVID-19 vaccine. Read the information carefully. We will ask you some questions about it.*" Then participants received information about the COVID-19 vaccine (for the full descriptions of both language conditions see Table S1).

Following the vaccine information and vaccine acceptance question (see full text), participants read, "*The Government (Hong Kong) is entering into agreements with vaccine developers of three vaccine candidates, namely BioNTech (BNT162b2), Sinovac Biotech (CoronaVac) and AstraZeneca (AZD1222). If you could choose any one of the three vaccines,*

which one would you select?" (1 = *BioNTech (BNT162b2)*, 2 = *Sinovac Biotech (CoronaVac)*, 3 = *AstraZeneca (AZD1222)*, 4 = *No preference*; all response options were presented in a random order for each participant). Following our main dependent variables (see full text), participants were asked a serious of questions of secondary interest (see Tables S2 to S4). At the end of the study, all participants were asked to translate the following sentences of the vaccine information from English to Chinese: (1) COVID-19 is very serious, and can cause severe health problems, and even death; (2) The vaccine helps protect you by strengthening your immune system; (3) Some people may have very mild side effects for a day or two after the vaccine. A professional translator, who was unaware of the language condition, research question and hypotheses, checked the accuracy of the translations.

Table S1

English and Chinese Materials Used in the Study

English	Chinese
**Why should I get the COVID-19 vaccine?**	**我為什麼要接種COVID-19疫苗？**
The COVID-19 vaccine is an important, safe and effective tool in the fight against the COVID-19 virus. COVID-19 is very serious, and can cause severe health problems, even death. The best way to protect yourself from COVID-19 is by getting the vaccine and taking other precautions, including practicing social distancing, continuing to wear a mask and washing your hands often and thoroughly.	COVID-19 疫苗是對抗 COVID-19 病毒的工具，既重要，亦安全有效。感染 COVID-19 的後果極為嚴重，能引致嚴峻的健康問題，更可能致死。保護自己免受 COVID-19 侵害的最佳方法是接種疫苗，並採取其他預防措施，包括保持社交距離，持續戴口罩及經常徹底洗手。
**How does the vaccine work?**	**疫苗如何發揮作用？**
The COVID-19 vaccine is delivered in two doses over the course of three to four weeks. The vaccine helps protect you by strengthening your immune system and helping it to recognize and fight the virus.	COVID-19 疫苗分兩劑接種，兩劑相隔三至四週。疫苗可增強免疫系統，並且識別及抵抗病毒，從而起保護作用。
**What are the possible side effects of the COVID-19 vaccine?**	**COVID-19 疫苗可能產生哪些副作用？**
Some people may have very mild side effects for a day or two after the vaccine. The side effects can be a sign that your body is developing an immune response to the vaccine. Common side effects include:<br>• redness, pain or swelling where the vaccine was given<br>• headache, feeling tired, muscle pain<br>• fever, chills, nausea, vomiting	有些人在接種疫苗後一兩天可能會有非常輕微的副作用。副作用可能表明您的身體正在對疫苗產生免疫反應。常見的副作用包括：<br>• 接種疫苗的位置發紅、疼痛或腫脹<br>• 頭痛、感覺疲倦、肌肉疼痛<br>• 發燒、發冷、噁心、嘔吐

Note. The information was originally written in English. We first translated the original materials into Chinese using a professional translation service. Then we back translated the Chinese translations into English using a professional translation service. Two independent native English bilinguals compared the original English and back translated English version. Two independent Chinese-English bilinguals corrected inconsistencies. Finally, two Chinese-English bilinguals checked the materials. In order to ensure that participants do not use the translation setting of their internet browser, we presented all information as image file format.

Table S2

Statistics of Variables

Variable	Chinese *M* (*SD*)	English *M* (*SD*)	Welch's *t*	*P*	*d*
Overall, how much do you trust that the COVID-19 vaccine will be effective?*[1]	**3.12 (0.97)**	**3.35 (0.81)**	**–3.25**	**.001**	**–0.26**
Overall, how much do you trust that the COVID-19 vaccine will be safe?*[1]	2.96 (0.94)	3.10 (0.85)	–1.94	.053	–0.16
How much do you worry about getting the COVID-19 vaccine?[2]	3.45 (1.06)	3.40 (1.02)	0.69	.493	0.06
How risky do you think it is to get the COVID-19 vaccine?[2]	3.41 (1.00)	3.30 (1.00)	1.52	.129	0.12
How beneficial do you think it is to get the COVID-19 vaccine?[2]	3.40 (0.98)	3.35 (0.06)	0.67	.501	0.06
What do you think is the probability of experiencing adverse side effects if you get the COVID-19 vaccine?*[1]	**4.40 (1.31)**	**4.06 (1.21)**	**3.35**	**< .001**	**0.27**
How serious do you think the possible side effects of the COVID-19 vaccine are?	4.63 (1.47)	4.45 (1.38)	1.51	.131	0.12
How confident are you that a COVID-19 vaccine will be adequately tested for safety and effectiveness?	3.96 (1.46)	3.86 (1.39)	0.93	.353	0.08
How much do you trust the COVID-19 vaccine information provided?*[1]	**4.66 (1.36)**	**4.32 (1.31)**	**3.12**	**.002**	**0.25**
How risky do you think an infection with COVID-19 is for your health?*[1]	**64.11 (23.19)**	**59.94 (24.68)**	**2.15**	**.032**	**0.17**
How severe do you think an infection with COVID-19 is for your health?*[1]	**4.96 (1.37)**	**4.56 (1.42)**	**3.52**	**< .001**	**0.29**
How much do you worry about getting infected with COVID-19?	4.38 (1.46)	4.24 (1.55)	1.20	.229	0.10
What is the probability that you will get COVID-19 as compared to other infectious diseases?	3.84 (1.16)	3.72 (1.20)	1.35	.177	0.11
If you do not get the COVID-19 vaccine and then get sick with COVID-19, how much would you regret not getting the vaccine?	3.05 (1.19)	3.04 (1.22)	0.05	.957	>0.01
If you do not get the COVID-19 vaccine and then get sick with COVID-19...how worried would you be about infecting your neighbours with COVID-19? [2]	3.37 (0.81)	3.29 (0.84)	1.25	.212	0.10
...how worried would you be about infecting your co-workers with COVID-19? [2]	2.60 (0.90)	2.60 (0.88)	–0.06	.950	–0.01
...how worried would you be about infecting your family members with COVID-19? [2]	2.76 (0.91)	2.89 (0.90)	1.74	.082	–0.14
...how worried would you be about infecting strangers with COVID-19?	2.53 (0.93)	2.44 (0.91)	1.28	.201	0.10

Note. Participants saw the variables in the designated language (Chinese or English). [1]Higher ratings indicate higher values in the dimensions of the scale. [2]The scales were accompanied by red or grey colors of increasing intensity. In order to ensure that participants do not use the default translation setting of their internet browser, we presented all information as image file format.

Table S3

Statistics of Exploratory Variables

Variable	Chinese *M* (*SD*)	English *M* (*SD*)	Welch's *t*	*P*	*d*
Most people who are important to me think that I should get vaccinated against COVID-19.	37.24 (27.14)	36.04 (27.27)	0.55	.590	0.04
It is expected of me that I will get vaccinated against COVID-19.	36.23 (25.28)	36.23 (25.79)	<0.08	1.00	<0.01
The people in my life whose opinions I value would want me to get vaccinated against COVID-19.	37.07 (28.22)	36.22 (28.46)	0.37	.711	0.03
How much do you know about how vaccines in general work?	64.06 (21.04)	63.80 (19.81)	0.16	.874	0.01
How much do you know about how vaccines in general are developed and tested?	53.22 (23.56)	52.67 (22.25)	0.30	.767	0.02
How severe was your case of the COVID-19?	4.00 (2.83)	1.20 (0.45)	1.39	.393	1.38
In general, would you say your health is:	2.90 (0.58)	2.89 (0.57)	0.28	.778	0.02
Compared to one year ago, how would you rate your health in general now?	2.99 (0.74)	2.89 (0.69)	1.74	.083	0.14
How often, if at all, do you get vaccinated against the annual seasonal flu?	4.57 (1.76)	4.44 (1.77)	0.88	.381	0.07
How often do you use natural home remedies instead of pharmaceuticals to try to prevent or cure illnesses?	**3.97 (1.04)**	**4.16 (1.00)**	**–2.36**	**.018**	**–0.19**
How strong is your sense of belonging to the Hong Kong community?	4.96 (1.48)	5.03 (1.50)	–0.54	.590	–0.04

Note. In both language conditions, these measures were presented in Chinese.

Table S4

Statistics of Exploratory Variables

Variable	Chinese %Yes	English %Yes	X^2	*P*	ϕ
Are you currently or have you been previously been infected with COVID-19?	0.6%	1.7%	1.61	.205	0.05
For statistical purposes only, do you have any of the following health conditions: asthma, cancer, chronic lung disease, diabetes, heart disease or a weakened immune system?	5.9%	8.9%	2.01	.157	–0.06

Note. In both language conditions, these measures were presented in Chinese.

Secondary Results

Vaccine Type Preference

Out of the 611 participants, 68.4% would choose BioNTech (BNT162b2), 7.2% Sinovac Biotech (CoronaVac), and 7.4% AstraZeneca (AZD1222), while 17.0% had no vaccine preference. Language did not influence vaccine type preference, Kruskal-Wallis $H(1) = 0.01$, $P = 0.945$.

Explaining the Language Effect on Vaccine Hesitancy through Trust and Worry

Research has shown that enhanced trust reduces negative feelings evoked by a novel avian flu vaccine and through this increases intentions to vaccinate[18]. Therefore, we tested a serial mediation model in which we evaluated whether language (0 = *Chinese*, 1 = *English*) influences vaccine hesitancy (0 = *Yes*, 1 = *Unsure/No*) through trust in the vaccine ("Overall, how much do you trust that the COVID-19 vaccine will be effective [safe]? 1 = *Do not trust at all to* 5 = *Completely trust*), which influences worry about the vaccine ("How much do you worry about getting the COVID-19 vaccine?" 1 = *Not at all worried* to 5 = *Very worried*) (see Figure S1 for the model). We controlled for age, gender, education and general health, yet the results remain similar when omitting these covariates from the model. We tested this model using 1,000 bootstrapping samples and computing the 95% confidence intervals.

There was a significant total indirect effect from language to trust to worry to vaccine hesitancy (total indirect effect: $b = –0.56$, 95% CI [–0.977, –0.218]). The serial indirect effect from language to trust to worry to vaccine hesitancy was also significant (serial indirect effect: $b = –0.07$, 95% CI [–0.147, –0.023]). Note the serial indirect effect is not significant if the order of trust and worry is reversed (serial indirect effect: $b = –0.09$, 95% CI [–0.245, 0.048]). The serial mediation path reflected three component paths, which were all significant: from language to trust ($b = 0.23$, 95% CI [0.099, 0.361]), from trust to worry ($b =$

–0.61, 95% CI [–0.692, –0.520]) and from worry to vaccine hesitancy (*b* = 0.52, 95% CI [0.271, 0.777]).

The effect of language on vaccine hesitancy was reduced when controlling for trust and worry (direct effect: *b* = –0.25, 95% CI [–0.685, 0.192]), consistent with full mediation. In sum, foreign English as compared to native Cantonese enhanced trust in the vaccine, which reduced worry associated with the vaccine, which in turn reduced vaccine hesitancy.

Figure S1

Language Effect on Vaccine Hesitancy Explained by Trust and Worry

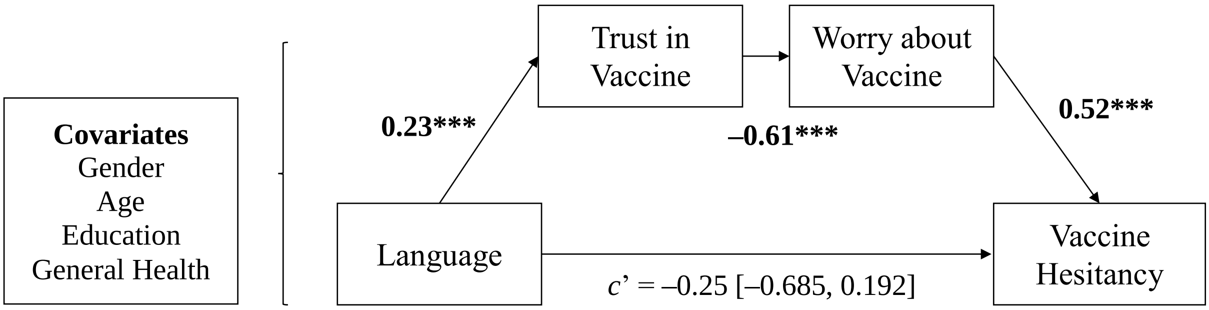

Note. Mediation coefficients represent unstandardized coefficients (95% CI in brackets).

Explaining the Language Effect on Vaccine Hesitancy through Trust, Worry and Risk/Benefit Perception

Research has shown that trust is a significant predictor influencing feelings evoked by products and through this the acceptance of products and vaccines[18,19,30]. Furthermore, feelings influence perceived benefit and risk of products[18,19,30]. Therefore, we tested a serial mediation model in which we evaluated whether language influences vaccine hesitancy through trust in the vaccine, which influences worry about the vaccine, which influences benefit/risk perception about the vaccine (we used the difference between benefit and risk). Figure S2 illustrates this serial mediation model. We controlled for age, gender, education and general health, yet the results remain similar when omitting these covariates from the

model. We tested this model using 1,000 bootstrapping samples and computing the 95% confidence intervals.

There was a significant total indirect effect from language to trust to worry to benefit–risk to vaccine hesitancy (total indirect effect: *b* = –0.50, 95% CI [–1.001, –0.108]). The serial indirect effect from language to trust to worry to benefit–risk to vaccine hesitancy was also significant (serial indirect effect: *b* = –0.07, 95% CI [–0.138, –0.028]). The serial mediation path reflected four component paths that were all significant: from language to trust, *b* = 0.23, 95% CI [0.099, 0.361]; from trust to worry, *b* = –0.61, 95% CI [–0.692, –0.520]; and from worry to benefit–risk, *b* = –0.64, 95% CI [–0.729, –0.545], and from benefit–risk to vaccine hesitancy, *b* = –0.82, 95% CI [–1.081, –0.565]. Note that the serial mediation is not significant if the order of trust and worry is reversed (indirect effect: *b* = –0.03, 95% CI [–0.088, 0.023]).

Figure S2

Language Effect on Vaccine Hesitancy Explained by Trust, Worry, and Risk-Benefit Index

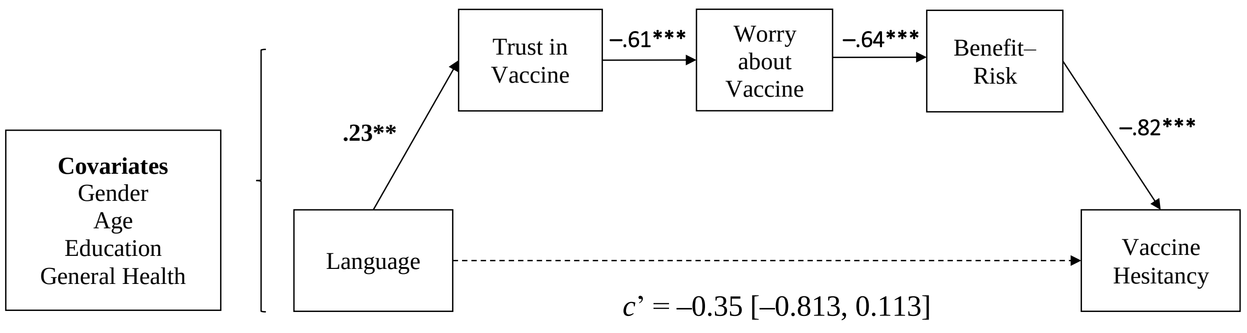

Note. Mediation coefficients represent unstandardized coefficients (95% CI in brackets).

The effect of language on vaccine hesitancy was significantly reduced when controlling for trust, worry, and benefit–risk (*b* = –0.35, 95% CI [–0.813, 0.113]), consistent with a full mediation. In sum, English as compared to Chinese increased trust in the vaccine, which

reduced worry associated with the vaccine, which reduced risk and increased benefit perception associated with the vaccine, which reduced vaccine hesitancy.
